# Phenotypical and functional characterization of a HepG2 cell clone stably overexpressing cytochrome P450 (CYP) 2C9

**DOI:** 10.1186/s13104-026-07935-y

**Published:** 2026-06-30

**Authors:** Sarah Kammerer, Natalie Herzog, Thea Jenchen, Valentina Stock, Rebecca Hofer, Veronika Ruzsanyi, Jan-Heiner Küpper

**Affiliations:** 1 Institute of Biotechnology, Brandenburg University of Technology Cottbus-Senftenberg, Universitätsplatz 1, 01968 Senftenberg, Germany; 2 Institute for Breath Research, Universität Innsbruck, Innrain 80/82, Innsbruck, 6020 Austria

**Keywords:** CYP2C9, HepG2, Cytochrome P450, Enzyme activity, Overexpressing cell line, Lentiviral transduction

## Abstract

**Objective:**

We aimed to generate a HepG2 cell clone stably overexpressing cytochrome P450 (CYP) 2C9 together with an empty vector (EV) control cell clone for pharmacological and toxicological studies of substances with CYP2C9-mediated biotransformation.

**Results:**

A new HepG2 cell clone was generated by lentiviral transduction to functionally overexpress human CYP2C9. We found high CYP2C9 transcript and protein levels based on qRT-PCR, Western blot and immunofluorescence in the cell clone. Most importantly, specific enzyme activities of 62.9 ± 2.6 pmol 4-hydroxydiclofenac/min/10^6^ cells as analyzed by diclofenac conversion via liquid chromatography-mass spectrometry were found in HepG2-CYP2C9 cells. CYP2C9 enzyme activity could successfully be inhibited by the application of the pan-CYP inhibitor 1-aminobenzotriazole or the CYP2C9-specific inhibitor sulfaphenazole. No changes in morphology or population doubling times were detected when comparing the clone to parental HepG2 cells. The corresponding EV control cell line was detected negative for CYP2C9 expression in all experimental setups and did not show any difference to the parental HepG2 cells. Thus, the newly established HepG2-CYP2C9 cell line is an appropriate tool to study metabolism and toxicity of substances depending on conversion by CYP2C9.

**Supplementary Information:**

The online version contains supplementary material available at 10.1186/s13104-026-07935-y.

## Introduction

Primary human hepatocytes (PHH) are regarded as the gold standard for in vitro toxicology studies, however, they are only limitedly available, show high donor variability, dedifferentiate quickly in culture and are thus difficult to use where reproducibility is important [1]. Due to these limitations, the human hepatoblastoma cell line HepG2 is extensively used as an alternative, owing to its unlimited availability and ease of use. Originating from the epithelial hepatoblastoma of a 15-year-old Caucasian male [2], HepG2 cells offer practical advantages but are limited by low or absent cytochrome P450 (CYP) oxidoreductase activity, which restricts their biotransformation capacity compared to PHH [3]. To address this limitation, we and others (e.g. [4, 5]). follow the approach of genetic modification of HepG2 cells. By overexpressing a single enzyme of interest, it becomes possible to study its dependent metabolism in a standardized cellular environment, while the latter is missing when isolated human liver microsomes (HLM) are used as in vitro model [6]. CYP2C9 makes up 20% of hepatic CYP proteins and metabolizes 15–20% of clinically used drugs [7, 8], underlining the importance of reliable CYP2C9 in vitro models. To test substrate specificities of tolterodine, diisopromine and the newly designed molecule gstachamine as potential candidates for unlabeled clinical CYP3A4 breath tests in different CYP overexpressing HepG2 cells [9–11], we thus developed a new HepG2 cell clone (HepG2-CYP2C9 clone 5) stably overexpressing human CYP2C9, alongside an empty vector (EV) control cell clone (HepG2-EV Zeo A1). While also others have generated CYP2C9 overexpressing HepG2 cells [4, 12], we aimed to provide full phenotypic and functional characterization including, next to mRNA and protein expression levels, also comparative population doubling times (PDT), enzyme activity levels and confirmative CYP(2C9) inhibition studies using different methods.

## Materials and methods

### Cell culture

HepG2 cells (HB-8065; ATCC, Manassas, USA) were maintained in Dulbecco’s Modified Eagle’s Medium (Biochrom AG, Berlin, Germany) supplemented with 10% fetal bovine serum (Biochrom AG) and 2 mM l-glutamine (PAA Laboratories GmbH, Pasching, Austria) at 37 °C with 5% CO₂ in a humidified incubator. Cells were routinely subcultured using 0.05% (w/v) trypsin/0.02% (w/v) EDTA (Biochrom AG). For all experiments presented here, cells were used in passages ranging from 17 to 35. Routine mycoplasma screening yielded negative results. Cell line authentication was performed on HepG2, HepG2-EV and HepG2-CYP2C9 cells by short tandem repeat profiling service (Eurofins Genomics, Ebersdorf, Germany) utilizing 16 DNA markers. Results authenticated all three samples as HepG2 cells (data available with the authors).

### Lentiviral infection of HepG2 cells

Plasmids were prepared with the Invitrogen™ Gateway^®^ Technology (Thermo Fisher Scientific, Waltham, MA, USA) according to the manufacturer’s protocol. The CYP2C9 coding sequence (NCBI reference sequence: NM_000771.5) in the resulting lentiviral expression vector was under control of the human cytomegalovirus promoter. The plasmid to produce EV controls did not contain the CYP2C9 sequence. The ViraPower™ Lentiviral Expression System (Thermo Fisher Scientific) was used for recombinant lentivirus production according to the manufacturer´s instruction. Briefly, 293FT cells were co-transfected with vector plasmid pLenti4/V5-DEST-CYP2C9 and helper plasmids pLP1, pLP2, pLP/VSVG via Lipofectamine™ 2000 (Thermo Fisher Scientific). Lentivirus particles were collected from supernatants after 48–72 h, 20-fold concentrated by filtration with the Vivaspin 20 system (Sartorius, Göttingen, Germany) and used directly for infection of 60% confluent HepG2 cells in 24 well plates (Sarstedt AG & Co. KG, Nümbrecht, Germany) with addition of 6 µg/ml hexadimethrine bromide (Sigma-Aldrich, Taufkirchen, Germany). Medium was replaced 24 h later with fresh HepG2 medium followed by start of selection pressure with 500 µg/ml zeocin (Genaxxon Bioscience, Ulm, Germany) 48 h post infection. After 3–4 weeks, zeocin-resistant colonies were isolated and expanded in zeocin containing medium to obtain single cell-derived HepG2 clones.

### Determination of population doubling times

Per well, 4 × 10^4^ cells were seeded into 6-well plates (Sarstedt AG & Co. KG) and cultured as described above without zeocin. Daily, cells were incubated for 15 min with 20 µg/ml Hoechst 33342 (Invitrogen, Darmstadt, Germany) for nuclear staining and 2 µg/ml propidium iodide (Invitrogen) to identify dead cells, followed by washing with phosphate buffered saline (PBS; Biochrom AG). Images (300 single images per well at 10x primary magnification) were taken using a BZ-X810 microscope (Keyence, Osaka, Japan) followed by image stitching and cell counting in *ImageJ* software (National Institutes of Health, Bethesda, MD, USA). PDTs were determined using the following formula, with t_2_−t_1_ = time interval of constant growth rate in hours, q_1_ = cell number per cm^2^ at t_1_, and q_2_ = cell number per cm^2^ at t_2_:

$$\:PDT=\:\frac{({t}_{2}-{t}_{1})\times\:log2}{\mathrm{l}\mathrm{o}\mathrm{g}\left(\frac{{q}_{2}}{{q}_{1}}\right)}$$

### Quantitative reverse transcriptase-polymerase chain reaction (qRT-PCR)

Total RNA was extracted from cell pellets with the innuPREP RNA Mini Kit (Analytik Jena AG, Jena, Germany) according to manufacturer’s instruction followed by 1% gel electrophoresis to check for RNA integrity. Oligo(dT)18 primers and RevertAid Reverse Transcriptase (both Thermo Fisher Scientific) were used for cDNA synthesis according to manufacturer’s protocol. Resulting cDNA was diluted 1:10 and subjected to qRT-PCR using the Maxima Probe qPCR Master Mix (Thermo Fisher Scientific), EvaGreen (Biotium, Freemont, CA, USA) and primer pairs (Biotez, Berlin, Germany; for sequences, see Supplementary Information 1) accordingly in a 10 µl reaction volume. Following settings on a C1000 Touch™ Thermal Cycler (Bio-Rad Laboratories, Inc., Hercules, USA) were used: initial denaturation: 95 °C for 3 min; 45 cycles: 95 °C for 10 s, 61 °C for 10 s and 72 °C for 30 s; final elongation: 72 °C for 30 s. As no *CYP2C9* Ct (threshold cycle) values were detected after 45 cycles for HepG2 and HepG2-EV cells, PCR products were visualized by 3% agarose gel electrophoresis instead performing relative quantification analysis by the comparative Ct method.

### Western blot

Protein was extracted from cell pellets by incubation with RIPA buffer containing protease inhibitor phenylmethylsulfonylfluoride (Sigma-Aldrich) for 15 min on ice followed by centrifugation (10,000×*g*, 10 min, 4 °C). Supernatants were subjected to protein concentration measurement using the Pierce BCA Protein Assay Kit (Thermo Fisher Scientific) according to manufacturer’s instructions. Samples were mixed with Laemmli buffer and heated for 5 min at 95 °C. For Western blot analysis, 25 µg total protein for HepG2 cells and 10 µg total protein of HLM (pooled from 50 individual donors; Thermo Fisher Scientific) were applied per lane for sodium dodecyl sulphate polyacrylamide gel electrophoresis followed by transfer to a PVDF membrane (Carl Roth GmbH + Co. KG, Karlsruhe, Germany). For the detection of CYP2C9 and the reference protein glyceraldehyde-3-phosphate dehydrogenase (GAPDH), anti-CYP2C9 monoclonal antibody (Thermo Fisher Scientific; dilution 1:500) and anti-GAPDH monoclonal antibody (Santa Cruz, Dallas, TX, USA; dilution 1:3000) were used as primary antibodies. Detection was performed with the ECL Select Kit (GE Healthcare, Freiburg, Germany) after incubation with horseradish peroxidase-conjugated secondary antibodies (Sigma-Aldrich). CYP2C9 band intensities were quantified in the 8-bit image type with *ImageJ* software and normalized to GAPDH band intensities. Results were expressed as target/reference ratio.

### Immunofluorescence staining

Per well, 4.5 × 10^4^ cells were plated in 96-well plates (Sarstedt AG & Co. KG) 24 h before fixation for 5 min with ice-cold methanol at room temperature followed by washing twice with PBS. CYP2C9 antibody (Thermo Fisher Scientific; dilution 1:150) was incubated over night at 4°C in 1.5% bovine serum albumin (BSA; Sigma-Aldrich) in PBS, followed by washing and incubation with Cy3-anti-mouse secondary antibody (Santa Cruz; dilution 1:200) together with DAPI (Carl Roth GmbH + Co. KG; 0.4 µg/ml) in 1.5% BSA in PBS for 1 h in the dark. After washing, cells were covered with PBS and images were taken with the fluorescence microscope BZ-X800 (Keyence) using same exposure settings across samples.

### Determination of enzyme activity by P450-Glo™ CYP2C9 assay

Per well, 4.5 × 10^4^ cells were plated in 96-well plates (Sarstedt AG & Co. KG) 24 h before beginning the P450-Glo™ CYP2C9 assay (Promega, Madison, WI, USA) according to the manufacturer’s instructions. Blank levels were measured by performing the assay in wells without cells. For inhibition assays, either 1 mM 1-aminobenzotriazole (1-ABT, Sigma-Aldrich; [13]) or 2 µM sulfaphenazole (SPZ, MedChemExpress, Monmouth Junction, NJ, USA; concentration as recommended by manufacturer) were added. Luminescence signals were measured using a FLUOstar Omega microplate reader (BMG Labtech, Ortenberg, Germany).

### Determination of enzyme activity by diclofenac conversion

Per well, 5 × 10^5^ cells were seeded in 24-well plates (Sarstedt AG & Co. KG) and cultured for 24 h. After washing with Krebs-Henseleit Buffer (KHB; completed with 25 mM NaHCO_3_, 25 mM HEPES, 2 mM CaCl_2_ × 2H_2_O; pH 7.4; all from Sigma-Aldrich except HEPES from Carl Roth GmbH + Co. KG), cells were incubated with 100 µM of CYP2C9-specific substrate diclofenac (Sigma-Aldrich) diluted in KHB (500 µl per well) for 4 h at 37 °C in a cell culture incubator. Blank levels were measured by performing the assay in wells without cells. For inhibition assays, 1 mM 1-ABT or 2 µM SPZ was included. Supernatants were then collected, mixed 1:1 with cold acetonitrile (Sigma-Aldrich), centrifuged at 13,000×*g* for 10 min and metabolite formation was measured by liquid chromatography-mass spectrometry (LC-MS). The analysis was performed using a Vanquish HPLC Flex system coupled with an Orbitrap Q-Exactive (Thermo Fisher Scientific). Separation was achieved using a ZORBAX Eclipse XDB-C18 column (1.8 μm, 2.1 × 100 mm), guarded by a ZORBAX Eclipse Plus C18 guard column (1.8 μm, 2.1 × 5 mm; both from Agilent Technologies, Santa Clara, CA, USA). The column temperature was set to 40 °C, while the temperature of the sample manager was held at 6 °C. For injections, 1 µL sample was used, and the eluents water (eluent A) and acetonitrile (eluent B) were each supplemented with formic acid 0.1% (v/v). The following gradient with a flow rate of 0.25 ml/min was used: 0–1 min 0% B; 1–5 min 0–90% B; 5–7 min 90% B; 7–7.2 min 90%–0% B; 7.2–9.2 min 0% B. The retention times of 4-hydroxydiclofenac and diclofenac were 5.38 min and 6.05 min, respectively. Mass spectrometric analysis was performed in positive ion mode, scanning a mass range of 50–500 amu, with a mass resolution of 35,000. The compounds were quantified using the corresponding [M+H]⁺ ions at *m/z* 296.02396 (diclofenac) and 312.01888 (4-hydroxydiclofenac). Data processing was conducted using QuanBrowser (Thermo Xcalibur 4.2.47, Thermo Fisher Scientific).

### Statistical analysis

GraphPad Prism 8.0 (GraphPad Software Inc., San Diego, CA, USA) was used for visualization and statistical analysis. One-way ANOVA with Tukey’s multiple comparison test was performed to compare groups. Results were considered statistically significant if *p* < 0.05. Data represent at least three independent experiments with technical duplicates or triplicates.

## Results

**Fig. 1:**
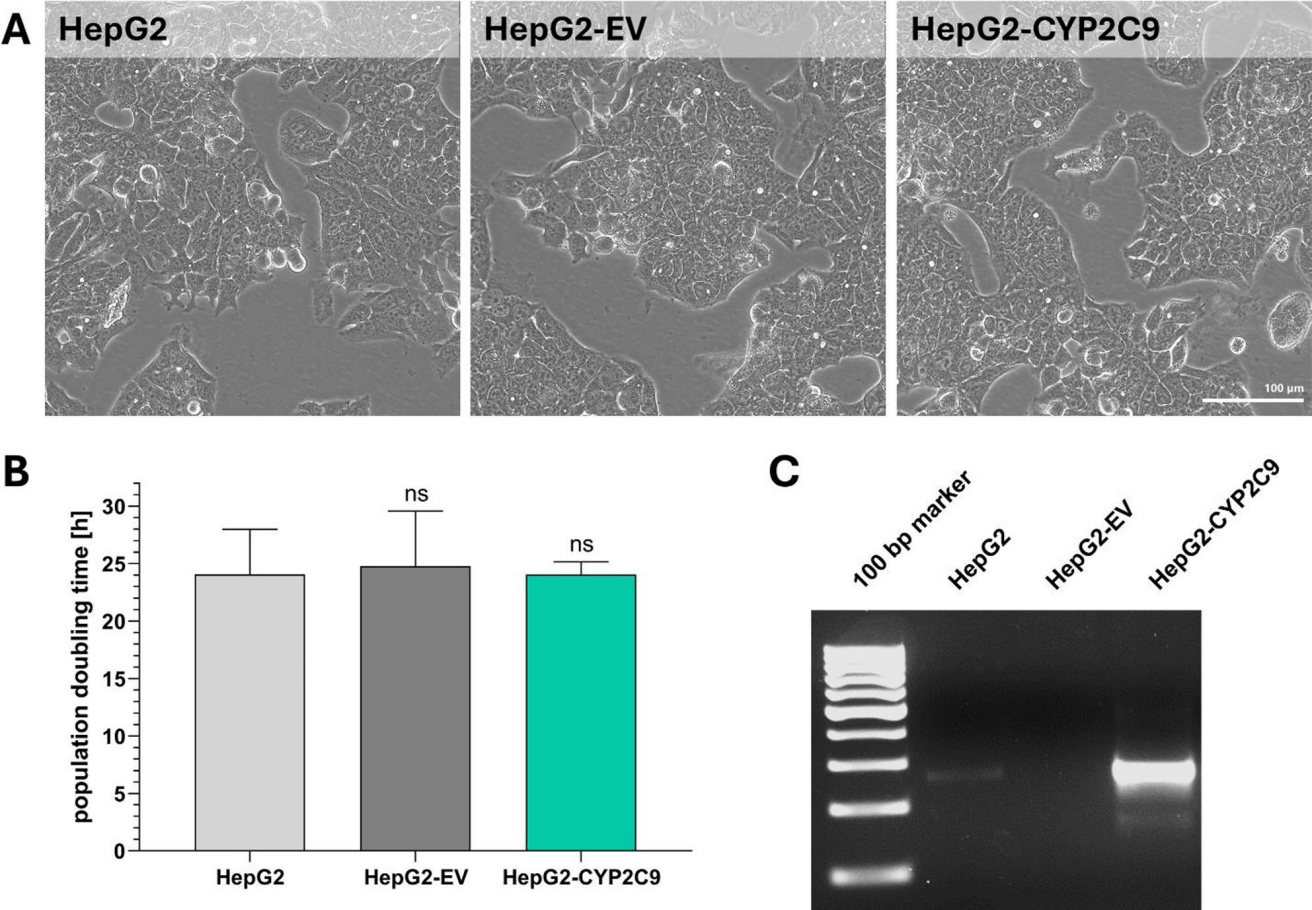
Phenotypic characterization of HepG2-CYP2C9 and -EV cells as compared to parental cells. **A** Phase contrast microscopy of parental HepG2, HepG2-EV and -CYP2C9 cells using a CKX41 Olympus microscope at 20x primary magnification. Representative images are shown. Scale bar: 100 μm. **B** Population doubling times of exponentially growing parental HepG2, HepG2-EV and -CYP2C9 cells. Results are shown as mean ± SD; ns: not significant. **C** Agarose gel electrophoresis of *CYP2C9* qRT-PCR products of parental HepG2, HepG2-EV and -CYP2C9 cells. bp: base pairs. Full gel images are shown in Supplementary Information 2

CYP2C9-overexpressing HepG2 alongside EV control cells were successfully generated via lentiviral transduction and single clone selection. Cells of HepG2 CYP2C9-overexpressing clone 5 (further designated HepG2-CYP2C9) and of HepG2 EV-overexpressing clone Zeo A1 (further designated HepG2-EV) displayed typical hepatocyte morphology (Fig. 1A) and exhibited similar PDTs (24.1 ± 1.1 and 24.8 ± 5.8 h, respectively) to those observed in parental HepG2 cells (24.1 ± 3.9 h; Fig. 1B). After 45 cycles, *CYP2C9* qRT-PCR was negative for parental HepG2 and HepG2-EV cells and thus, PCR products were visualized by agarose gel electrophoresis instead performing relative quantification analysis by the comparative Ct method. Gel electrophoresis for *CYP2C9* PCR products showed a weakly recognizable band at the correct size for parental HepG2 cells and a strong specific signal for HepG2-CYP2C9 cells (Fig. 1C and Supplementary Information 2). We further screened the samples to test whether the lentiviral transduction would have influenced the expression of other relevant hepatic genes. A qRT-PCR on nuclear receptor *CAR*, seven CYP enzymes (*CYP1A2*, -*2B6*, -*2C8*, -*2C9*, -*2C19*, -*2E1* and − *3A4*) and three phase II enzymes (*GSTA1*, *GSTP1* and *UGT1A1*) indicated that most of the genes were not expressed in HepG2 cells. A difference in Ct-values was only found for CYP2C9 which was clearly detectable in HepG2-CYP2C9 cells but not in parental and EV HepG2 cells (Supplementary Information 3).

**Fig. 2:**
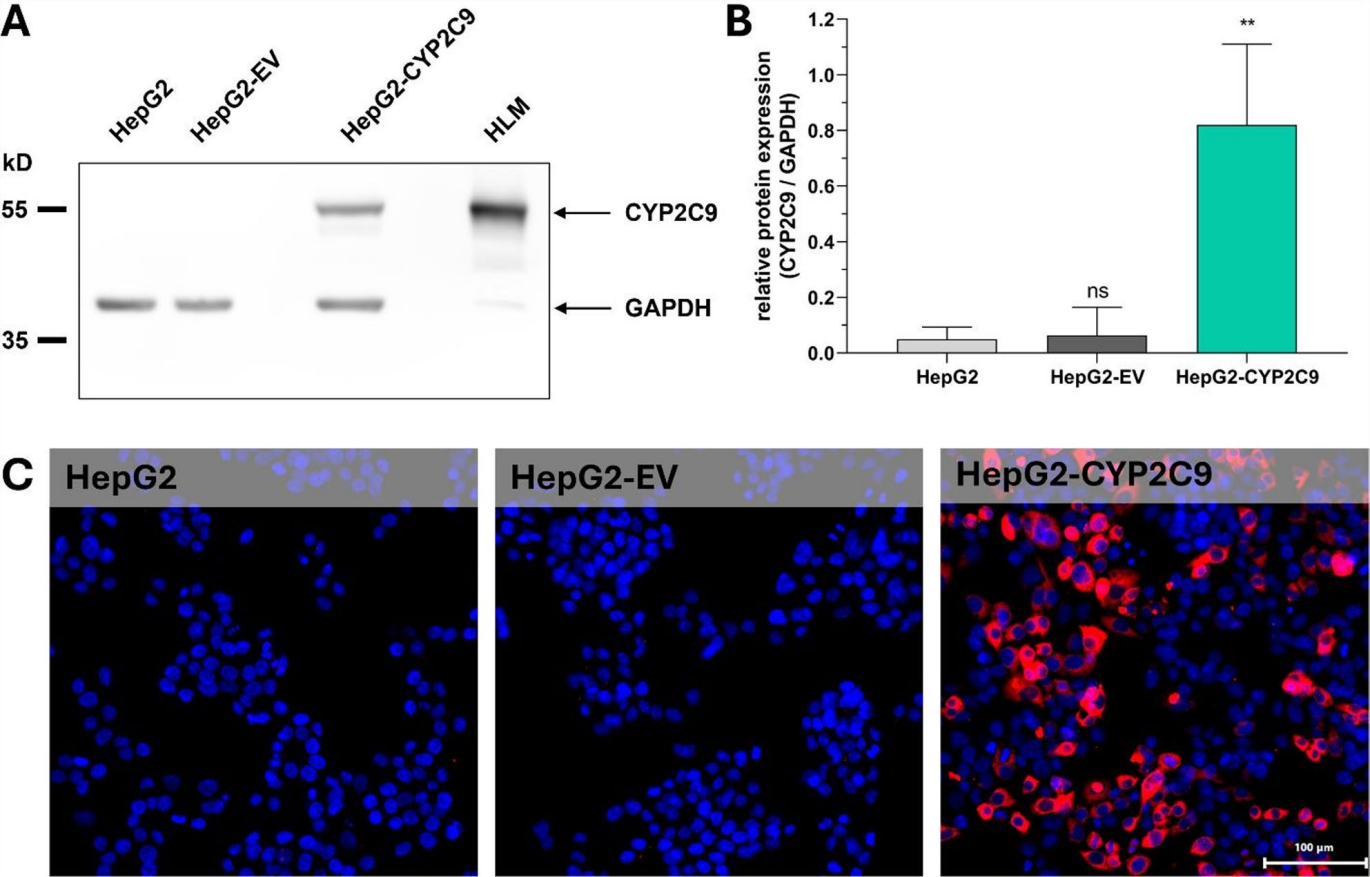
CYP2C9 protein expression in HepG2-CYP2C9 and -EV cells as compared to parental cells. **A** Western blot analysis of parental HepG2, HepG2-EV and -CYP2C9 cells. Pooled human liver microsomes (HLM) served as positive control and GAPDH served as loading control. Note that microsomal protein extracts do not contain GAPDH. Representative image is shown. Full membrane images are shown in Supplementary Information 4. **B** Quantification of Western blot band intensities. Relative protein expression is shown as mean ± SD; ns: not significant; **p < 0.01. **C** Immunofluorescence staining of CYP2C9 (shown in red) counterstained by DAPI (cell nuclei shown in blue) of parental HepG2, HepG2-EV and -CYP2C9 cells. Images were taken with a Keyence microscope at 20x primary magnification. Representative images are shown. Scale bar: 100 μm

Next, we aimed to confirm overexpression of CYP2C9 also at protein level. As Western blot data demonstrate, parental HepG2 and HepG2-EV cells did not harbor detectable amounts of CYP2C9 protein, while CYP2C9-overexpressing HepG2 cells displayed strong and specific signals (Fig. 2A and Supplementary Information 4). Quantification of Western blot band intensities showed negligible signals for control cells, but a CYP2C9:GAPDH ratio of 0.82 ± 0.29 for HepG2-CYP2C9 (Fig. 2B). Immunofluorescence staining further confirmed the absence of CYP2C9 protein in parental HepG2 and HepG2-EV cells and strong CYP2C9 expression in HepG2-CYP2C9 (Fig. 2C).

**Fig. 3:**
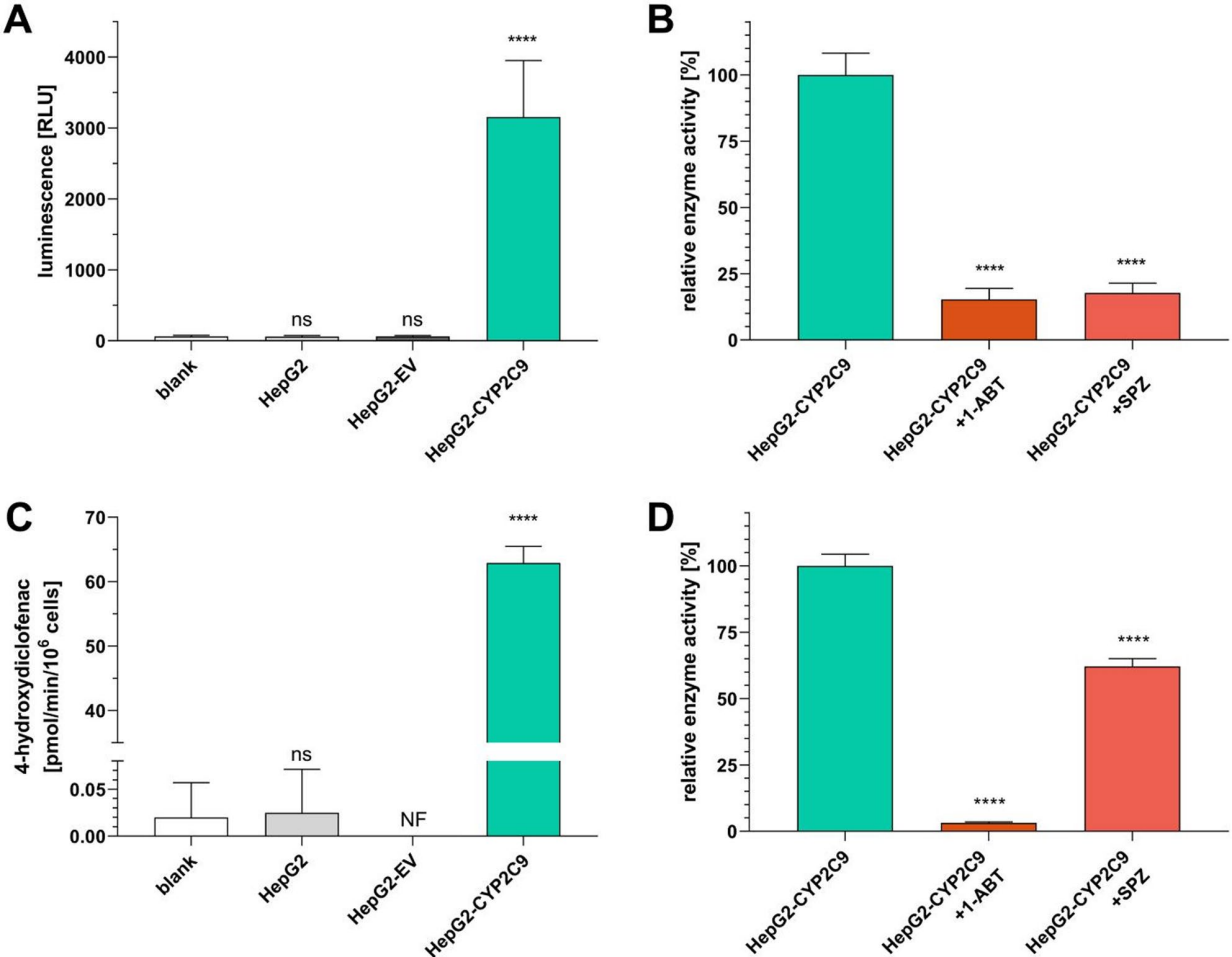
Functional characterization of CYP2C9 in HepG2-CYP2C9 and -EV cells as compared to parental cells. **A** Relative luminescence units (RLU) in parental HepG2, HepG2-EV and -CYP2C9 cells as compared to blank levels (wells without cells) measured by the P450-Glo™ CYP2C9 assay. **B** Relative enzyme activity of HepG2-CYP2C9 cells after treatment with inhibitors 1-aminobenzotriazole (1-ABT) or sulfaphenazole (SPZ) measured by the P450-Glo™ CYP2C9 assay. **C** Determination of enzyme activity in parental HepG2, HepG2-EV and -CYP2C9 cells as compared to blank levels (wells without cells) by conversion of diclofenac to 4-hydroxydiclofenac measured by LC-MS. Note the interruption of the y-axis from 1 to 35 for better visualization of low values. **D** Relative enzyme activity of HepG2-CYP2C9 cells after treatment with inhibitors 1-ABT or SPZ measured by conversion of diclofenac to 4-hydroxydiclofenac via LC-MS. All results are shown as mean ± SD; NF: not found; ns: not significant, ****p < 0.0001

Finally, functionality of CYP2C9 was assessed through enzyme activity assays. First, using the P450-Glo™ CYP2C9 assay, it was proven that parental and HepG2-EV cells did not display any measurable CYP2C9 enzyme activity, as relative luminescence units were at blank levels (wells incubated without cells) for these cells. In contrast, and as expected from protein expression levels, HepG2-CYP2C9 cells showed significant enzyme activities (Fig. 3A). To further confirm the specificity of the reaction, two different CYP inhibitors were employed on HepG2-CYP2C9 cells, namely the pan-specific mechanism-based irreversible CYP inhibitor 1-ABT and the CYP2C9-specific competitive inhibitor SPZ. Application of both inhibitors led to a similar and significant reduction to 15.3 ± 4.2 and 17.7 ± 3.7% residual enzyme activities as measured by the P450-Glo™ CYP2C9 assay, respectively (Fig. 3B). Second, CYP2C9-specific activity was confirmed by diclofenac conversion to 4-hydroxydiclofenac via LC-MS measurement. Metabolite production ranged at blank levels for parental HepG2 cells and could not be detected at all for HepG2-EV cells. Recombinant CYP2C9 expression in HepG2-CYP2C9 cells yielded 62.9 ± 2.6 pmol 4-hydroxydiclofenac/minute/10⁶ cells (Fig. 3C). Again, an inhibition assay with 1-ABT and SPZ was performed with HepG2-CYP2C9 cells and measured by LC-MS. Addition of 1-ABT resulted in almost complete enzyme inhibition with a residual CYP2C9 activity of only 3.17 ± 0.41%. SPZ treatment resulted in a lesser but still highly significant inhibition with 62.1 ± 3.0% residual enzyme activity (Fig. 3D).

## Discussion

Here, we present a full phenotypic and functional characterisation of a newly generated HepG2 cell line overexpressing CYP2C9. We could prove that the parental and the corresponding EV control cells contained, if any, only low CYP2C9 levels in all experimental setups, whereas CYP2C9 expression and activity were high in HepG2-CYP2C9 cells. This expression can be regarded stable for at least 18 passages as no significant changes in CYP2C9 expression or enzyme activity levels were obvious in a range from passage 17 to 35 as used in the results presented here. HepG2-CYP2C9 cells, together with other CYP overexpressing HepG2 cells, were already successfully used to study whether tolterodine, diisopromine or the newly synthesized molecule gstachamine would be substrates for distinct CYP enzymes [9–11]. Most importantly, a clear-cut CYP2C9 enzyme activity was detected in our newly established HepG2-CYP2C9 clone with a diclofenac to 4-hydroxydiclofenac conversion activity of 62.9 pmol/min/10^6^ cells. However, direct comparison of these activity levels to those reported in literature remains difficult as others used tolbutamide instead of diclofenac as prototypical CYP2C9 substrate (e.g [14]). or they defined enzyme activity in alternative units (mainly pmol/min/mg protein). Given the prerequisite that there is a linear correlation between total protein and cell number in cultured liver cells with approximately 300 pg protein/cell [15], we found literature values for PHH ranging from 1.5 to 123.3 pmol/min/10^6^ cells regarding their 4-hydroxylase activity on diclofenac [16, 17]. This underlies the highly donor-dependent nature of CYP2C9 activity in humans. Our cell model thus does not range at the upper end of possible CYP2C9 activity levels measured in PHH. However, it might be regarded as model representing the median physiological activity found in human liver. Also others have previously generated CYP2C9 overexpressing HepG2 cells by lentiviral transduction [4, 12]. However, CYP2C9 enzyme activity levels are not directly presented in these studies which makes it difficult to compare these cells with our results and with enzyme activity in PHH. Further, no confirmatory CYP inhibition experiments were performed in these previous studies. Our inhibition experiments successfully proved specificity of observed reactions, however, CYP2C9 activity was only inhibited by about 40% by CYP2C9-specific inhibitor sulfaphenazole in our LC-MS based diclofenac conversion experiment while almost complete inhibition could be achieved with this inhibitor when using the P450 CYP2C9 Glo™ assay. This discrepancy could be explained by the different modes of action of the inhibitors [13, 18] together with unequal seeding densities of cells for the P450-Glo™ CYP2C9 and the LC-MS assay. With regard to surface area, about 140,000 cells/cm^2^ were seeded for the P450-Glo™ assay in 96 well plates, while about 260,000 cells/cm^2^ were used for diclofenac incubation in 24 well plates followed by LC-MS measurement. Cell densities were previously optimized for each assay, however, this imbalance is likely accounting for the incomplete CYP2C9 inhibition seen with SPZ in the LC-MS assay, where more cells per surface area were used. Contribution of other CYP enzymes to diclofenac conversion is considered highly unlikely due to the fact that the substrate is specific to CYP2C9 [19]. It should be noted that diclofenac can also undergo 5-hydroxylation via CYP3A4 [20], albeit to a much lesser extent. As our LC-MS does not allow a clear-cut separation of 4- and 5-hydroxydiclofenac, we cannot completely exclude the production of 5-hydroxydiclofenac in our setting. Despite basal CYP3A4 activity has been reported in the literature [21], we could not detect *CYP3A4* mRNA expression in our cells and therefore, we regard the production of 5-hydroxydiclofenac as unlikely. In summary, we have generated a CYP2C9 overexpressing HepG2 cell clone that, together with its EV control cell line, can be a valuable tool for toxicological and pharmacological studies of CYP2C9-dependant substrates.

### Limitations

HepG2 cells are known to display only low basal to negative CYP enzyme expression levels [22]. Genetic manipulation is thus required to produce HepG2 cells with stable and defined CYP overexpression for their use in toxicological and pharmacological research. However, other relevant drug-metabolizing CYP enzymes remain absent and a complete hepatic differentiation profile cannot be achieved by such approaches. Furthermore, also the tumorigenic origin of HepG2 cells may hinder physiological studies dependant on the research question. PHH or primary-like liver cells such as upcyte hepatocytes remain a better choice for studies requiring full hepatic metabolism competence [23].

## Supplementary Information

Below is the link to the electronic supplementary material.

Supplementary Material 1.

## Data Availability

The datasets used and/or analysed during the current study are available from the corresponding author on reasonable request.

## Abbreviations

1-ABT: 1-Aminobenzotriazole
BSA: Bovine serum albumin
Ct: Threshold cycle
CYP: Cytochrome P450
EV: Empty vector
GAPDH: Glyceraldehyde-3-phosphate dehydrogenase
HLM: Human liver microsomes
KHB: Krebs-Henseleit Buffer
LC–MS: Liquid chromatography–mass spectrometry
NF: Not found
ns: Not significant
PBS: Phosphate buffered saline
PDT: Population doubling time
PHH: Primary human hepatocytes
qRT-PCR: Quantitative real-time polymerase chain reaction
SD: Standard deviation
SPZ: Sulfaphenazole
