# Supplementary material for: Phenotypical and functional characterization of a HepG2 cell clone stably overexpressing cytochrome P450 (CYP) 2C9
Source: BMC Res Notes. 2026 Jun 30;19:264. doi: 10.1186/s13104-026-07935-y (PMC13321531; doi:10.1186/s13104-026-07935-y)
Supplement: Supplementary file 1 — Supplementary Material 1. [file 13104_2026_7935_MOESM1_ESM.pdf]

Supplementary Information 1

Primer sequences used for qRT-PCR.

	**forward sequence (5'→3')**	**reverse sequence (5'→3')**
CAR	CGGATCAGCTCTTCTTGCTC	ATGCTGGCATGAGGAAAGAC
CYP1A2	CCAGCTGTCAGATGAGAAG	CCTGCCAATCACTGTGTC
CYP2B6	CTCTCCATGACCCACACTAC	TGTTGGGGGTATTTTGCCCA
CYP2C8	CTTCCGTGCTACATGATGAC	CCTTCTCCTGCACAAATTCG
CYP2C9	CCAACCCAGAGATGTTTGAC	GAATGAAGCACAGCTGGTAG
CYP2C19	TTGACCCTCGTCACTTTCTG	GTTGTGTCAAGGTCCTTTGG
CYP2E1	TGGATGCTGTGGTGCATGAG	GAGTCCAGAGTTGGCACTAC
CYP3A4	GTGGGGCTTTTATGATGGTCA	GCCTCAGATTTCTCACCAACACA
GSTA1	CAAGCTCCTCGACGTAGTAG	ATCCTCCTTCTGCCCGTATG
GSTP1	CGGGCAAGGATGACTATGTG	GCTAGGACCTCATGGATCAG
UGT1A1	CTCTCCTCTCATTCAGATCAC	CAAAGTCACTTCTAAACAGCC
GAPDH	TGCACCACCAACTGCTTAGC	GGCATGGACTGTGGTCATGAG

Supplementary Information 2

Full unprocessed gel images of PCR products, referring to Figure 1C. Each gel image represents one independent run of the PCR. The white dashed boxes indicate the gel edges. The green dashed boxes indicate the relevant samples (*CYP2C9* product of HepG2, HepG2-EV, HepG2-CYP2C9) and the orange dashed box in replicate #3 indicates the cropped image used in Figure 1C.

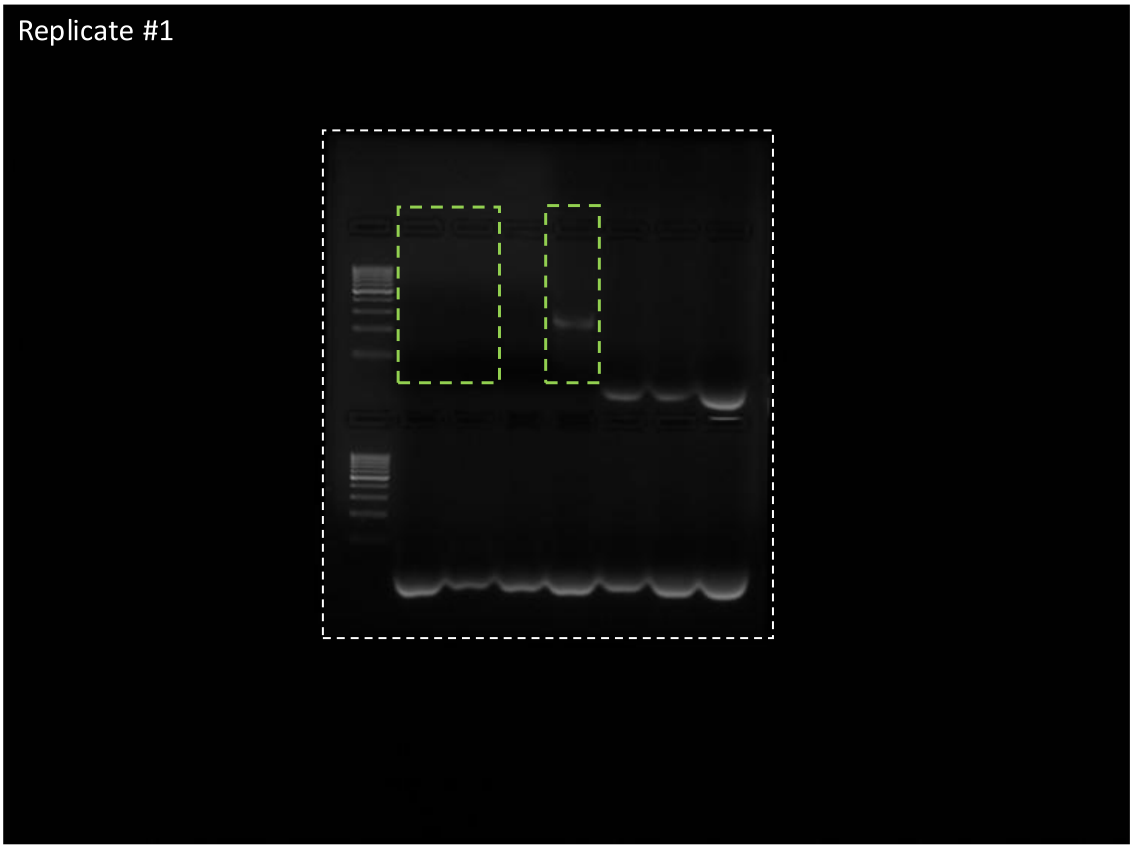

Lanes (top): 1=100 bp DNA ladder; 2=CYP2C9 product of HepG2; 3=CYP2C9 product of HepG2-EV Zeo A1; 4=CYP2C9 product of HepG2-EV Zeo A2; 5=CYP2C9 product of HepG2-CYP2C9; 6=CYP2C9 NTC; 7=GAPDH product of HepG2; 8=GAPDH product of HepG2-EV Zeo A1.

Lanes (bottom): 1=100 bp DNA ladder; 2=GAPDH product of HepG2; 3=GAPDH product of HepG2-EV Zeo A1; 4=GAPDH product of HepG2-EV Zeo A2; 5=GAPDH product of HepG2-CYP2C9; 6=CYP2C9 NTC; 7=GAPDH product of HepG2-EV Zeo A2; 8=GAPDH product of HepG2-CYP2C9.

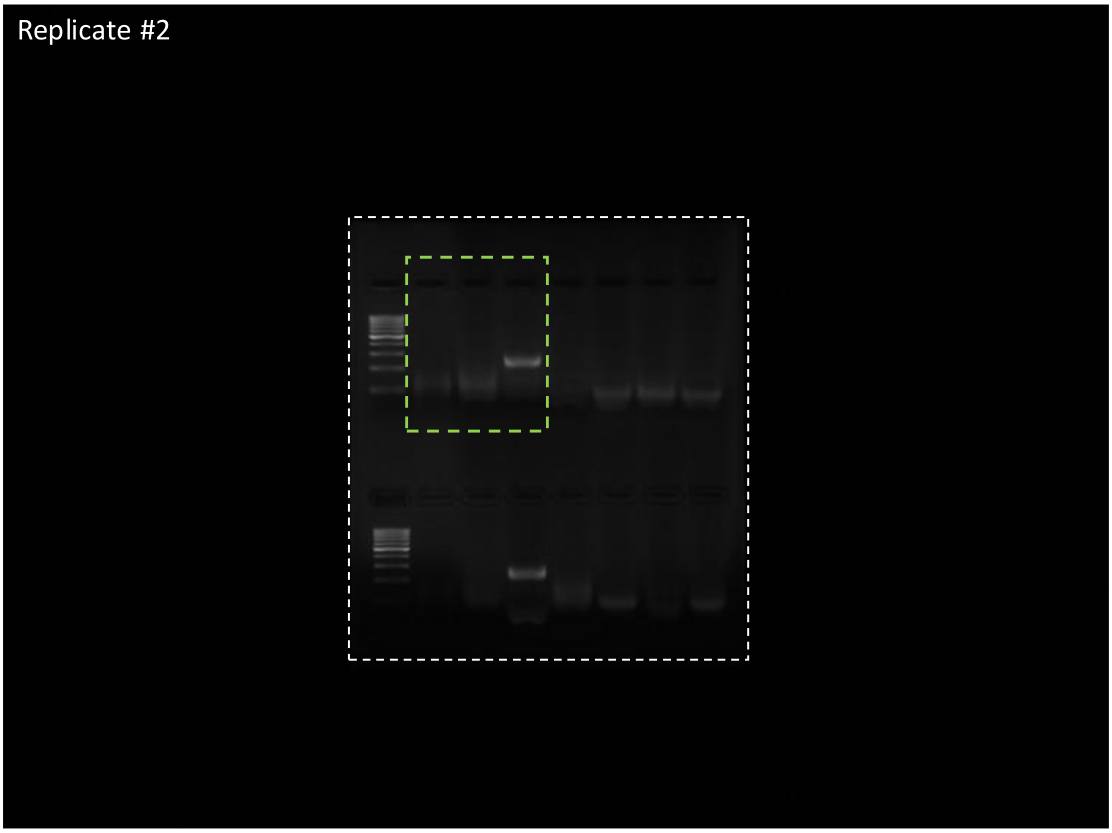

Lanes (top): 1=100 bp DNA ladder; 2=CYP2C9 product of HepG2; 3=CYP2C9 product of HepG2-EV Zeo A1; 4=CYP2C9 product of HepG2-CYP2C9; 5=CYP2C9 product of HepG2-EV Zeo A3; 6=GAPDH product of HepG2; 7=GAPDH product of HepG2-EV Zeo A1; 8=GAPDH product of HepG2-EV Zeo A3.

Lanes (bottom): 1=100 bp DNA ladder; 2=CYP2C9 product of HepG2; 3=CYP2C9 product of HepG2-EV Zeo A3; 4=CYP2C9 product of HepG2-CYP2C9; 5=CYP2C9 product of HepG2-EV Zeo A1; 6=GAPDH product of HepG2-CYP2C9; 7=CYP2C9 NTC; 8=CYP2C9 NTC.

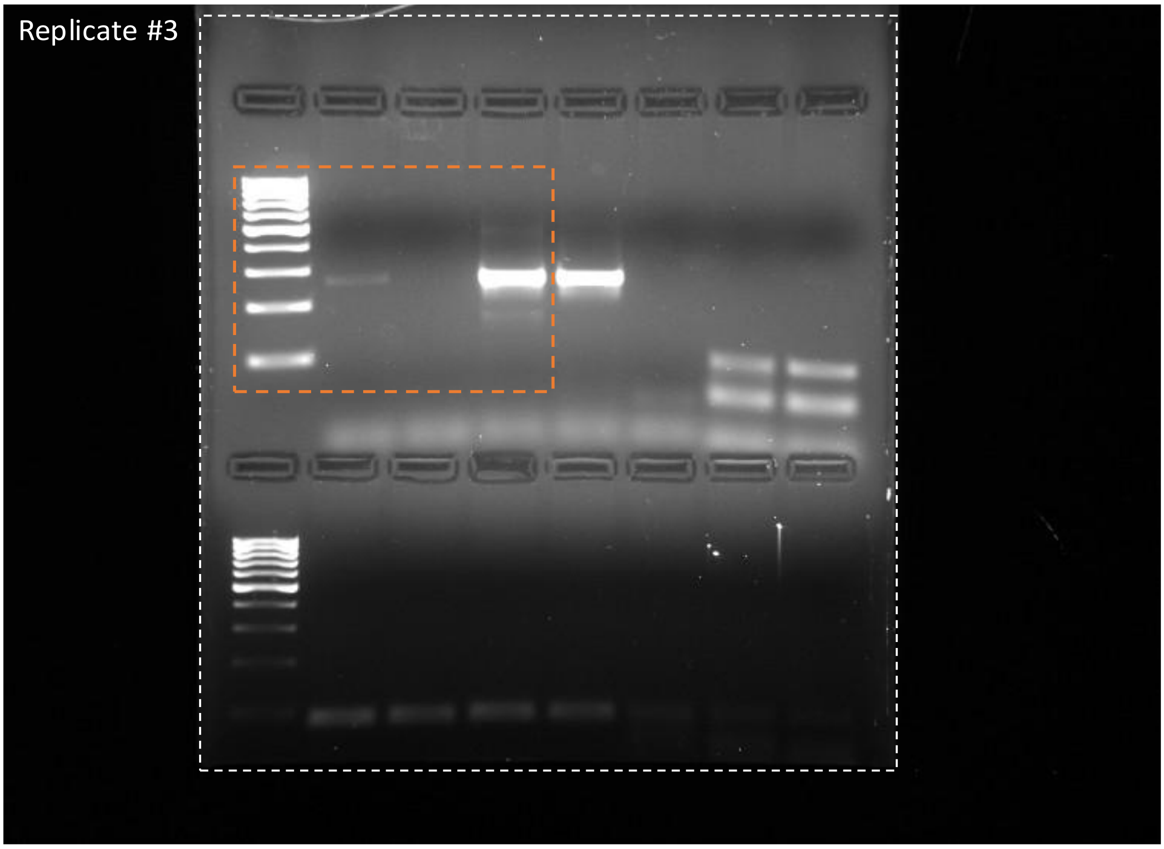

Lanes (top): 1=100 bp DNA ladder; 2=CYP2C9 product of HepG2; 3=CYP2C9 product of HepG2-EV Zeo A1; 4=CYP2C9 product of HepG2-CYP2C9; 5=CYP2C9 product of pooled primary human hepatocytes; 6=CYP2C9 NTC; 7=GAPDH product of HepG2 -RT; 8= GAPDH product of HepG2-EV Zeo A1 –RT.

Lanes (bottom): 1=100 bp DNA ladder; 2=GAPDH product of HepG2; 3=GAPDH product of HepG2-EV Zeo A1; 4=GAPDH product of HepG2-CYP2C9; 5=GAPDH product of pooled primary human hepatocytes; 6=GAPDH NTC; 7=GAPDH product of HepG2-CYP2C9 -RT; 8= GAPDH product of pooled primary human hepatocytes –RT.

Supplementary Information 3

A qRT-PCR was performed to screen HepG2, HepG2-EV and HepG2-CYP2C9 cells for the nuclear receptor *CAR*, seven phase I enzymes (*CYP1A2*, *-2B6*, *-2C8*, *-2C9*, *-2C19*, *-2E1*, and *-3A4*), three phase II enzymes (*GSTA1*, *GSTP1* and *UGT1A1*) and reference gene *GAPDH* as described in the main text, but with 40 cycles. The results are given as Ct (threshold cycle)-values (mean ± SD, n=3) in the table below (*NF: not found within 40 cycles*).

	**HepG2**	**HepG2-EV**	**HepG2-CYP2C9**
CAR	NF	NF	NF
CYP1A2	NF	NF	NF
CYP2B6	NF	NF	NF
CYP2C8	NF	NF	NF
CYP2C9	NF	NF	**29.0 ± 0.6**
CYP2C19	NF	NF	NF
CYP2E1	NF	NF	NF
CYP3A4	NF	NF	NF
GSTA1	34.5 ± 0.7	34.7 ± 0.3	35.3 ± 0.1
GSTP1	NF	NF	NF
UGT1A1	37.4 ± 1.1	37.3 ± 0.0	37.33 ± 0.1
GAPDH	20.5 ± 1.2	21.3 ± 0.9	20.2 ± 0.8

Supplementary Information 4

Full unprocessed images of Western blot membrane strips, referring to Figure 2A. Each membrane image represents one independent replicate run of the Western blot. The black dashed boxes indicate the membrane edges after re-alignment for image acquisition.

After blotting, the membranes were cut into two strips between the 35 and the 55 kD marker bands for separate antibody incubation of CYP2C9 and GAPDH (upper and lower strip, respectively). The membranes were further trimmed above the 70 kD marker band and below the 35 kD marker band to fit the membrane strips into the incubation chambers. The membrane strips were processed as described and then re-aligned for image acquisition; the blue line indicates both the cut position and the re-alignment boundary between the two strips. The green dashed box in replicate #3 indicates the cropped image used in Figure 2A.

Replicate #1

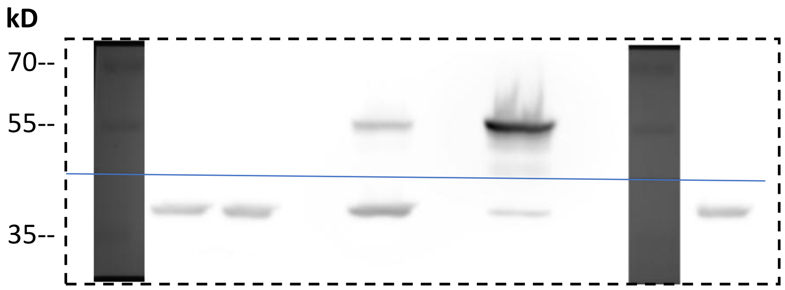

Top membrane strip: CYP2C9 staining; bottom membrane strip: GAPDH staining.

Lanes: 1=protein size marker; 2=HepG2; 3=HepG2-EV Zeo A1; 4=empty; 5=HepG2-CYP2C9; 6=empty; 7=pooled human liver microsomes; 8=empty; 9=protein size marker; 10=HepG2-CYP2C19.

Replicate #2

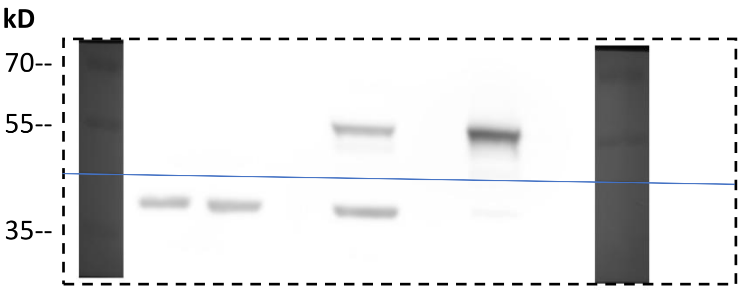

Top membrane strip: CYP2C9 staining; bottom membrane strip: GAPDH staining.

Lanes: 1=protein size marker; 2=HepG2; 3=HepG2-EV Zeo A1; 4=empty; 5=HepG2-CYP2C9; 6=empty; 7=pooled human liver microsomes; 8=empty; 9=protein size marker; 10=empty.

Replicate #3

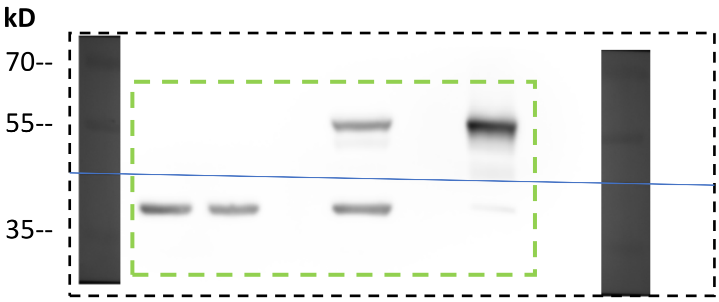

Top membrane strip: CYP2C9 staining; bottom membrane strip: GAPDH staining.

Lanes: 1=protein size marker; 2=HepG2; 3=HepG2-EV Zeo A1; 4=empty; 5=HepG2-CYP2C9; 6=empty; 7=pooled human liver microsomes; 8=empty; 9=protein size marker; 10=empty.
